# The effects of urine alkalinization on kidney function in critically ill patients with COVID-19: a proof-of-concept randomized clinical trial

**DOI:** 10.1186/s40635-025-00739-7

**Published:** 2025-03-07

**Authors:** Nuttha Lumlertgul, John A. Kellum, Jonah Powell-Tuck, Moncy Mathew, Sunita Sardiwal, Marlies Ostermann

**Affiliations:** 1 Division of Nephrology, Excellence Center in Critical Care Nephrology, King Chulalongkorn Memorial Hospital, Bangkok, Thailand; 2 Faculty of Medicine, Chulalongkorn University, Bangkok, Thailand; 3 Center of Excellence for Critical Care Nephrology, Department of Critical Care Medicine, University of Pittsburgh, Pittsburgh, PA USA; 4 Department of Critical Care, King’S College London, Guy’S & St Thomas’ NHS Foundation Hospital, London, SE1 7EH UK; 5 Pharmacy Department, Guy’S & St Thomas’ NHS Foundation Hospital, London, UK; 6 Department of Biochemistry, Guy’S & St Thomas’ NHS Foundation Hospital, London, UK

**Keywords:** COVID-19, AKI, Urinary alkalinization, SARS-CoV-2, Acute kidney injury

## Abstract

**Background:**

Acute kidney injury (AKI) is a common complication of COVID-19. While the exact mechanisms remain unclear, direct viral infection of renal tubular epithelial cells is hypothesized. Given the pH-dependent entry of coronaviruses into host cells, urine alkalinization was proposed as a potential preventive strategy.

**Methods:**

This was a proof-of-concept prospective, randomized clinical trial in critically ill patients with COVID-19. Patients were randomized to urine alkalinization versus usual care. The intervention group received intravenous 8.4% sodium bicarbonate to achieve a urine pH ≥ 7.5 up to 10 days after randomization. The primary outcome was the proportion of patients achieving target urine pH. Secondary outcomes included changes in urine tissue inhibitor of metalloproteinases-2 (TIMP-2) and insulin-like growth factor-binding protein 7 (IGFBP7), AKI development, renal replacement therapy, and adverse effects.

**Results:**

The trial was terminated early due to slow recruitment and the end of the COVID-19 pandemic. Sixteen patients were enrolled (median age 48 years old, 75% male). More patients in the intervention group achieved target urine pH than in the control group (75% vs 37.5%, *P* = 0.315). There was a separation of urine pH between both groups throughout 10 days (*P* = 0.097 for interaction). However, the intervention did not significantly impact urine [TIMP-2]x[IGFBP7] concentrations (*P* = 0.813 for interaction) or clinical outcomes, including AKI occurrence (risk ratio 0.6 (95% confidence interval 0.21, 1.70), *P* = 0.619). More patients in the intervention group experienced hypernatremia and metabolic alkalosis. Notably, patients with elevated urine [TIMP-2]x[IGFBP7] concentrations and AKI had higher ICU and 60-day mortality.

**Conclusions:**

While urine alkalinization is feasible and can increase urine pH, we could not demonstrate differences in AKI rates or changes in urine [TIMP-2]x[IGFBP7] concentrations in critically ill COVID-19 patients.

**Supplementary Information:**

The online version contains supplementary material available at 10.1186/s40635-025-00739-7.

## Background

Severe Acute Respiratory Syndrome Coronavirus 2 (SARS-CoV-2) infection results in Coronavirus Disease 2019 (COVID-19) with a vast range of symptoms and organ involvement. Although COVID-19 primarily affects the lungs, other organs, including the gastrointestinal tract, heart, and kidney, may be involved. Acute kidney injury (AKI) secondary to COVID-19 (COV-AKI) occurs in 15–25% of patients hospitalized with COVID-19 and up to 46–77% of intensive care unit (ICU) patients [1, 2]. The majority of AKI cases are mild to moderate with renal replacement therapy (RRT) prescribed in 3–32% of patients [3, 4]. Similar to other etiologies, COV-AKI is associated with increased risks of short-term and long-term mortality and deterioration of kidney function [1, 5].

Multiple pathophysiologic mechanisms contribute to the development of COV-AKI, such as hemodynamic instability, inflammation, endothelial dysfunction, and indirect effects from therapies [6–9]. However, AKI might also result from direct infection of renal tubule epithelial cells (RTEC). A variety of epithelial cells express the angiotensin-converting enzyme-2 (ACE2) receptor, which is used by β-coronaviruses to enter the cells [10, 11]. Viral genetic material has been demonstrated in renal tissues of COVID-19-affected subjects, and the virus has been recovered from the urine [12–15].

SARS-CoV-2 shares many of the characteristics of other coronaviruses. SARS-CoV-2 infects human body cells through several key steps. Initially, the virion binds to the cell membrane. Second, the S1 subunit dissociates upon cleavage by host proteases, e.g., transmembrane protease serine 2 (TMPRSS2) which leads to viral membrane fusion allowing the genome to penetrate the cytosol [16]. The docking and membrane fusion processes require an acidic environment at pH 6.2–6.8 [17, 18]. Therefore, blocking the virus from entering the cells may be a potential target to prevent severe COVID-19.

Coronavirus infectivity is pH and temperature dependent. Coronaviruses are stable at pH 6.0 and 37 °C (half-life 24 h), but conformational changes occur at pH 8.0 and 37 °C affecting function [19]. Consequently, changes in pH may affect the receptor binding domain (RBD) of β-coronaviruses S1 subunit to bind to ACE2 receptor and infectivity [20].

Urine pH is normally 5.5–6.5, but can be manipulated. Urine alkalinization protocols using intravenous sodium bicarbonate solution are prescribed in several medical situations, e.g., to reduce renal toxicity from salicylate and phenobarbital intoxication, during methotrexate treatment and following exposure to uranyl compounds [21].

It was our hypothesis that urine alkalinization may inhibit the virus from infecting RTEC. We aimed to explore the feasibility and safety of urine alkalinization in critically ill patients with COVID-19 to prevent COV-AKI.

## Materials and methods

### Study design and setting

This was an investigator-initiated, prospective, parallel-group, open-label feasibility randomized-controlled trial (RCT) conducted between July 2021 and January 2022 at Guy’s & St Thomas’ Hospital, a public University Hospital in the National Health Service (NHS) in the United Kingdom. The protocol was approved by the Health Research Authority (HRA), Research Ethics Committee (REC), and the Medicines and Healthcare Products Regulatory Agency (MHRA) for Clinical Trial Authorization. The study was prospectively registered on the European Union Drug Regulating Authorities Clinical Trials Database (EudraCT number 2020–004934-39) and on the ClinicalTrials.gov database (NCT04655716). All procedures were conducted in accordance with the ethical standards of the responsible committee on human experimentation and the Helsinki Declaration of 2024.

### Patients

The following inclusion criteria were applied: 1) confirmed SARS-CoV-2 positive; 2) admission to an ICU; 3) bladder catheter in situ; 4) central venous line in place; and 5) age ≥ 18 years. Exclusion criteria included: 1) stage 3 AKI as defined by Kidney Diseases: Improving Global Outcomes (KDIGO) criteria [22]; 2) chronic kidney disease stage 4 or 5; 3) contraindications to sodium bicarbonate therapy; 4) serum sodium > 150 mmol/L; 5) blood pressure > 180/100 mmHg; 6) severe hypokalemia (K < 3.0 mmol/L); 7) severe hypocalcemia (ionized calcium < 0.8 mmol/L); 8) urine pH > 7.5; 9) pregnant or lactating women; and 10) on medications that may interact with sodium bicarbonate. After eligibility was confirmed, written informed consent was sought from the patient. If the patient did not have capacity to consent, we consulted with their legal representative or an independent professional consultee. As soon as the patient had regained capacity, consent for continuation was sought.

### Randomization

Enrolled patients were randomly allocated to the intervention or control group on a 1:1 basis using sequentially numbered, opaque, sealed envelopes.

### Intervention

In the intervention group, sodium bicarbonate solution was administered to achieve a target urine pH of 7.5–8.5. Sodium bicarbonate 8.4% was given as an initial 1 ml/kg intravenous infusion, followed by 0.5 ml/kg infusions at 10-min intervals until a total of 225 ml had been administered. The urine pH was checked 30 min after each bolus. Repeat boluses of intravenous sodium bicarbonate 8.4% at 0.5 ml/kg were administered if necessary to achieve the target urine pH of 7.5–8.5 as long as the total volume of sodium bicarbonate 8.4% did not exceed 900 ml in 24 h. After the pH target was achieved, urine pH was checked every 12 h. Urine alkalinization continued for up to 10 days or until the patient was discharged from the ICU or until the primary endpoint was reached. All other aspects of care were determined by the clinical team, including the use of additional fluid therapy, electrolyte supplementation, vasopressors, and organ support modalities. Diuretics could be administered to control fluid balance, and acetazolamide could be used to correct fluid overload and metabolic alkalosis. Patients assigned to the control group received usual care as determined by the clinical team. In the control group, urine pH was checked every 12 h until the patient was discharged from the ICU.

### Outcomes

The feasibility outcomes included: 1) total volume of NaHCO₃ 8.4% administered in 24 h and over the study period; 2) the number of NaHCO₃ boluses; and 3) time to achieve urine pH ≥ 7.5 (days). The primary endpoint was the proportion of patients who achieved urine pH ≥ 7.5 over the duration of the intervention. Secondary outcomes were: 1) changes in urine tissue inhibitor of metalloproteinases-2 (TIMP-2) and insulin-like growth factor-binding protein 7 (IGFBP7) concentrations over 10 days; 2) development and maximal stage of AKI (according to KDIGO criteria) within 10 days after randomization; 3) receipt of RRT in the first 28 days; 4) cumulative vasopressor-free days at day 28; 5) RRT-free days at day 28; 6) mechanical ventilation-free days at day 28; 7) PaO₂/FiO₂ ratio at days 3, 7, and 10; 8) change in Sequential Organ Failure Assessment (SOFA) score at day 3 after randomization; 9) cumulative fluid balance at days 3, 7, and 10; 10) ICU and hospital length of stay; and 11) ICU, hospital, 28-day, and 60-day mortality. Safety outcomes included: 1) hypernatremia (serum sodium > 150 mmol/L); 2) hypokalemia (K < 3.0 mmol/L); 3) hypocalcemia (ionized calcium < 0.8 mmol/L); and 4) metabolic alkalosis (serum pH > 7.50).

### Laboratory measurement

Urine pH was measured regularly by dipstick. Arterial blood gases and electrolytes were measured at baseline and at regular intervals. Urine samples were collected at baseline and daily up to 10 days after randomization to measure urine TIMP-2 and IGFBP7 concentrations using VIDAS^®^ NEPHROCHECK^®^ laboratory method. VIDAS^®^ NEPHROCHECK^®^ is an automated test for the immunoenzymatic quantitative determination of TIMP-2 and IGFBP7 proteins in human urine using the Enzyme Linked Fluorescent Assay (ELFA) technique.

### Statistical analysis

The primary clinical outcome ‘proportions of patients achieving urine pH ≥ 7.5’ was used for sample size calculation. In the absence of representative data, the estimate 50% difference of the proportions was based on the previous literature in salicylate poisoning. Using a power of 80%, a two-sided alpha of 0.05, and a 10% drop-off rate, 42 patients (21 per group) were required.

We performed the primary analysis on the intention-to-treat (ITT) population defined as all randomized subjects with consent to use data in the analysis. Categorical variables are reported as absolute numbers and percentages, and compared using Fisher’s exact tests. Continuous variables are reported as median (quartile 25th–quartile 75th) and compared using Wilcoxon rank-sum tests or Kruskal–Wallis test. Continuous outcomes were compared between the groups using median difference with 95% confidence interval (CI) calculated after median regression models with bootstrapping. Categorical outcomes were compared between the groups using risk ratio (RR) calculated from a generalized linear model with a binomial distribution and reported with its 95% CI. Continuous variables over time were compared between the groups using a mixed effect generalized linear model with Gaussian distribution, and with intervention, time (as continuous variable) and intervention × time interaction added as fixed effect, and with patients added as random effect to account for repeated measurements. All analyses were performed with Stata 18.0 (StataCorp, Texas, USA). A p < 0.05 was considered statistically significant.

## Results

### Baseline characteristics

**Fig. 1:**
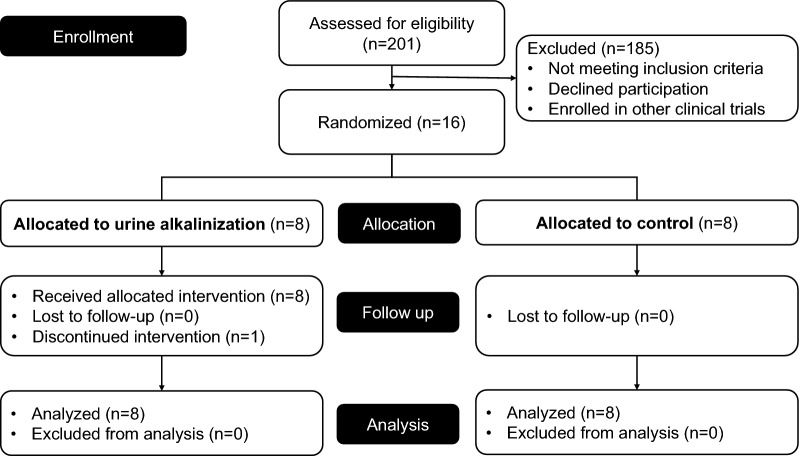
Study flow

Recruitment to the study was discontinued after enrollment of 16 patients as the number of patients with COVID-19 requiring ICU admission was rapidly decreasing at the end of the COVID-19 pandemic. Of the 16 patients enrolled, 8 were randomized to the urine alkalinization group and 8 to the usual care group (Fig. 1). Baseline characteristics were well balanced between the treatment groups (Table 1). The median age was 47 [interquartile range (IQR) 40–61] years, 12 (75%) were male. Baseline pH, sodium concentration, and base excess (BE) results were comparable between both groups. Baseline urine pH was 5 (IQR 5–5). All patients were invasively ventilated, and 50% were on vasopressor support.

**Table 1:** Baseline characteristics

	Intervention (n = 8)	Control (n = 8)
Age (years)	42 (38.5, 61)	56 (41.5, 61.5)
Male sex	5 (62.5)	7 (87.5)
BMI [kg/m^2^]	29.86 (25.25, 33.46)	27.41 (27.36, 27.76)
Chronic comorbidities		
Hypertension	4 (50)	2 (25)
Diabetes	5 (62.5)	0
COPD	1 (12.5)	1 (12.5)
CKD	0	1 (12.5)
Peripheral vascular disease	0	1 (12.5)
Malignancy	1 (12.5)	0
ACEIs/ARBs	2 (25.0)	1 (12.5)
Diuretics	0	0
NSAIDs	1 (12.5)	1 (12.5)
Baseline SCr [µmol/L]	81 (74, 84)	100 (77, 104)
SCr at first hospital admission [µmol/L]	91.5 (71.5, 113)	116 (77.5, 143)
Parameters at randomization		
Days between ICU admission and randomization	4 (2.5, 6)	3.5 (1, 6)
APACHE II	10 (6, 13.5)	13 (9.5, 20)
Total SOFA score	5 (3.5, 7)	8 (6.5, 9.5)
Laboratory results		
Creatinine [µmol/L]	88 (73, 107.5)	82 (69.5, 133.5)
pH	7.37 (7.34, 7.45)	7.40 (7.36, 7.43)
Base excess	−0.3 (−1.6, 2.05)	0.4 (−1.6, 3.9)
Sodium [mmol/L]	144 (140, 146)	142 (141, 143)
Urine pH	5 (5, 5)	5 (5, 5)
Lactate [mmol/L]	2.55 (2.3, 3.25)	2.65 (2.2, 3.25)
CRP [mg/L]	23.5 (4, 43.5)	18.5 (10.5, 47.5)
Albumin [g/dL]	29 (28.5, 33.5)	31 (30.5, 35)
Hb [g/dL]	110.5 (103, 117.5)	125.5 (117.5, 129)
WBC [× 10^9^/L]	12.1 (10.35, 16.75)	7.95 (3.73, 14.9)
Platelet [× 10^9^/L]	279.5 (187, 379.5)	157.5 (113, 239)
PaO_2_/FiO_2_ ratio	141.95 (122.08, 179.92)	117.56 (90.82, 135.58)
PaCO_2_ [kPa]	5.25 (4.93, 6.32)	5.99 (5.6, 6.28)
Organ support		
Mechanical ventilation	8 (100)	8 (100)
Vasopressor	2 (25)	6 (75)
Treatment for COVID-19		
Steroids	7 (87.5)	5 (62.5)
Antiviral therapy	2 (25)	2 (25)

*ACEIs* angiotensin-converting enzyme inhibitors, *APACHE II*, Acute Physiology and Chronic Health Evaluation II, *ARBs* angiotensin receptor blockers, *BMI* body mass index, CKD chronic kidney disease, *COPD* chronic obstructive pulmonary disease, *COVID-19* coronavirus disease 2019, *CRP* c-reactive protein, *Hb* hemoglobin, *ICU* intensive care units, *NSAIDs* non-steroidal anti-inflammatory drugs, *SCr* serum creatinine, *SOFA* Sequential Organ Failure Assessment, *WBC* white blood cells

### Intervention

**Fig. 2:**
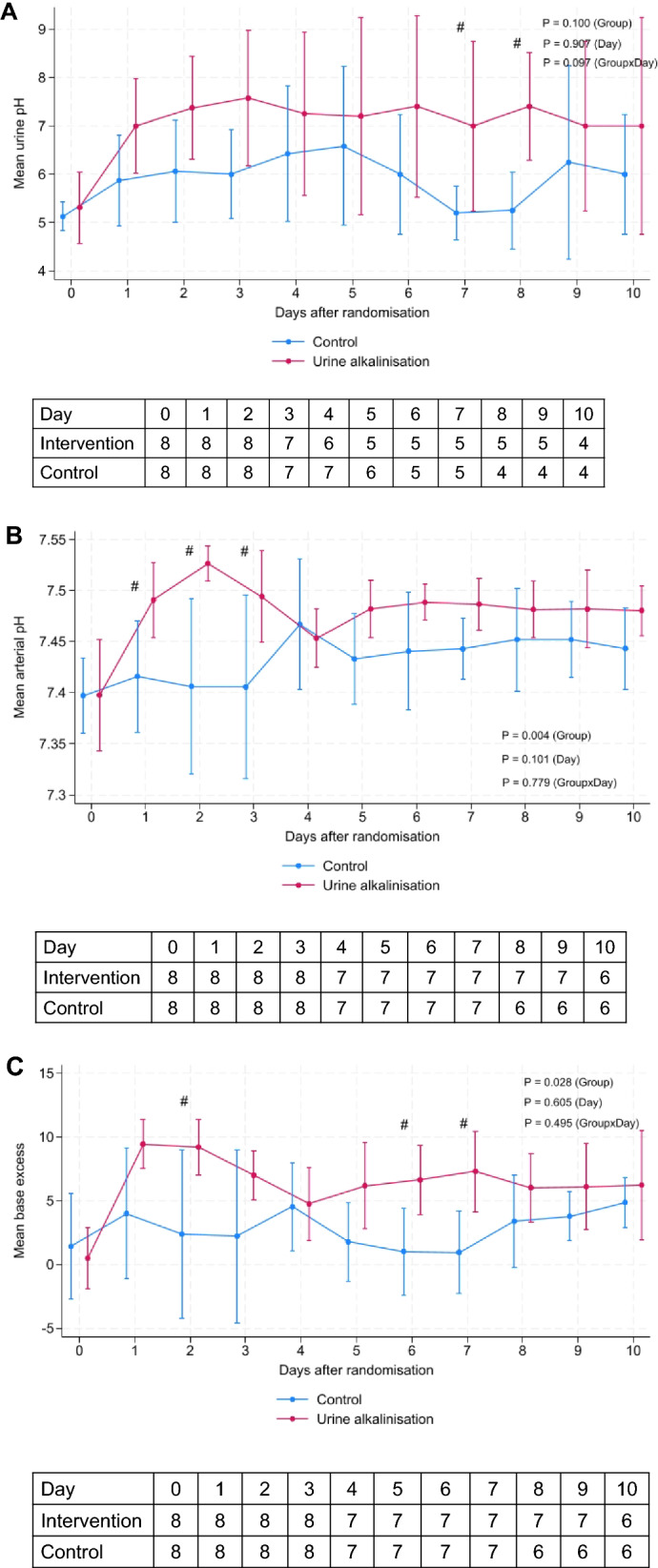

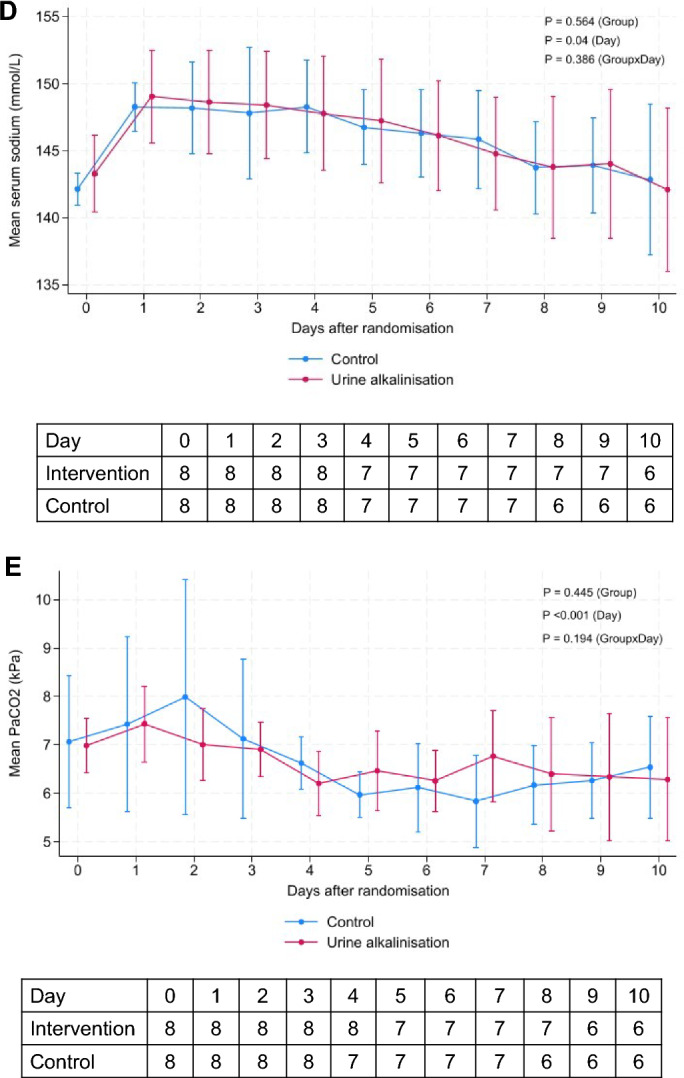
Changes in urine pH (**A**), arterial pH (**B**), base excess (**C**), sodium (**D**), and PaCO_2_ (**E**) concentrations between the urine alkalinization group and usual care during 10 days of the study period

Patients in the urine alkalinization group received a median of 225 (IQR 225–305) mL within the first 24 h and 2600 (IQR 2600–2600) mL over the study period. The median number of bicarbonate boluses was 11 (IQR 2–16). More patients in the intervention group achieved target urine pH than in the control group (75% vs 37.5%), but this difference was not significant (P = 0.315). The proportions of measurements with urine pH ≥ 7.5 were significantly higher in the intervention group than the usual care group (32.5% vs 6%, *p* < 0.0001). There was an increase in urine pH from baseline in the intervention group compared with the usual care group (P = 0.097 for interaction) (Fig. 2A). The median time to achieve urine pH ≥ 7.5 was 20 (IQR 10, 27) h. During 10 days of follow-up, arterial pH and BE results were higher in the intervention group (Fig. 2B–D). Sodium concentrations increased from baseline in both groups and peaked at day 1. Serum PaCO_2_ values decreased significantly over time, but there were no differences between the groups (Fig. 2E). Potassium and ionized calcium concentrations were lower in the intervention group than in the control group (Table 2).

**Table 2:** Biochemical outcomes

	Intervention (n = 8)	Control (n = 8)	P value
Total volume of NaHCO_3_ 8.4% received during study period [ml]	2600 (2600, 2600)	–	–
Volume of NaHCO_3_ 8.4% received in 24 h [ml]	225 (225, 305)	–	–
Number of NaHCO_3_ 8.4% boluses	11 (2, 16)	–	–
Total number of urine pH measurements per patient	37 (13, 44)	21 (16, 29)	0.226
Time to achieve urine pH ≥ 7.5 (hours)	20 (10, 27)	48 (6, 56)	0.697
Number of patients achieving urine pH ≥ 7.5 (%)	6 (75)	3 (37.5)	0.315
Proportions of urine pH ≥ 7.5 of all urine pH measurements (%)	32.5	6.02	< 0.0001
Number of blood gas analyses during study period	115 (97, 120)	104 (82, 118)	0.777
Serum pH			
- lowest	7.26 (7.11, 7.32)	7.32 (7.14, 7.33)	< 0.001
- highest	7.56 (7.53, 7.77)	7.49 (7.48, 7.53)	< 0.001
Base excess			
- lowest	−0.8 (−3.4, −0.5)	−2.2 (−5.9, −0.9)	< 0.001
- highest	12.4 (11.8, 13.1)	5.9 (2.2, 10.4)	< 0.001
PaCO_2_ [kPa]			
- lowest	4.37 (3.91, 4.66)	4.32 (4.00, 4.65)	0.92
- highest	7.84 (7.55, 8.35)	7.48 (7.25, 8.23)	0.40
Serum bicarbonate [mmol/L]			
- lowest	24 (22, 25)	22 (18, 23)	< 0.001
- highest	38 (36, 38)	31 (28, 35)	< 0.001
Serum lactate [mmol/L]			
- lowest	2.15 (1.8, 2.3)	1.45 (1.25, 2.1)	0.315
- highest	4.15 (3.65, 4.6)	3.95 (3.65, 5.65)	0.858
Serum sodium [mmol/L]			
- lowest	136 (133, 143)	140 (135, 141)	< 0.001
- highest	153 (145, 153)	150 (148, 152)	< 0.001
Serum ionized calcium [mmol/L]			
- lowest	1.04 (1.03, 1.1)	1.06 (1.04, 1.07)	0.0001
- highest	1.32 (1.24, 1.43)	1.29 (1.28, 1.39)	0.937
Serum potassium [mmol/L]			
- lowest	3.53 (3.18, 3.59)	3.905 (3.72, 4.1)	< 0.001
- highest	5.46 (5.4, 5.67)	5.81 (5.51, 6.34)	< 0.001

### Clinical outcomes

**Fig. 3:**
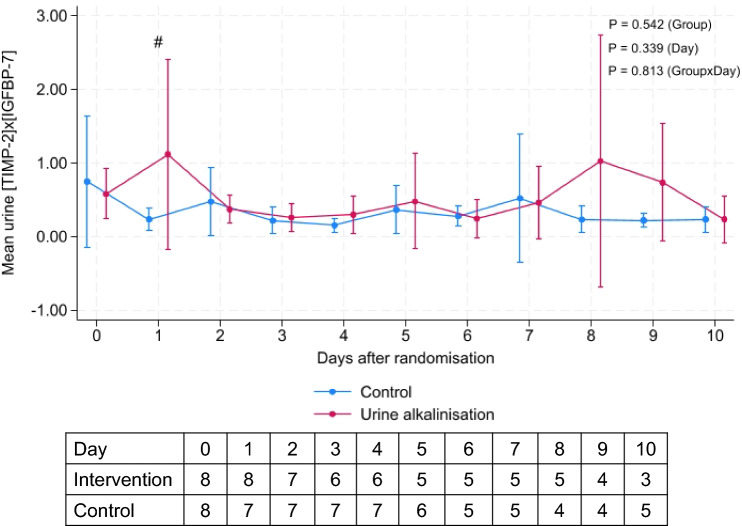
Changes in urine [TIMP-2]x[IGFBP7] between the urine alkalinization and placebo groups

Ten days after randomization, there were no differences in changes of urine [TIMP-2]x[IGFBP7] results between the urine alkalinization and the placebo groups (*P* = 0.81 for interaction) (Fig. 3). Three patients (37.5%) in the intervention group and 6 (62.5%) in the control group developed AKI [RR 0.6 (95% CI 0.21, 1.70), *p* = 0.62]. The median onset date of AKI was 3 (IQR 1, 6) days. Patients in the intervention group were more likely to receive diuretics including furosemide and bendroflumethiazide than the usual care group. All other secondary outcomes, including PaO_2_/FiO_2_ ratio, cumulative fluid balance, change in SOFA score, ICU, and hospital lengths of stay and 60-day mortality,were similar between the groups (Table 3).

**Table 3:** Clinical outcomes

	Intervention (n = 8)	Control (n = 8)	Intervention vs control (95% CI)	P value
Any stage AKI	3 (37.5)	5 (62.5)	0.6 (0.21, 1.70)	0.619
AKI onset (days)	4 (1, 5)	1 (1, 6)	3 (−3, 9)	1.000
AKI stage 1	3 (37.5)	2 (25.0)	1.07 (0.27, 4.23)	0.500
AKI stage 2	0	1 (12.5)	0	
AKI stage 3	0	2 (25.0)	0	
RRT	0	2 (25.0)	0	0.467
Proportion of patients receiving diuretic therapy	8 (100)	6 (75)	0	0.467
Days on diuretics	8 (5, 11)	4 (1, 6)	4 (−2.95, 10.95)	0.073
Total furosemide dose during study period (mg)	150 (40, 240)	35 (5, 80)	120 (−57.31, 297.31)	0.065
Total bendroflumethiazide dose during study period (mg)	16.25 (7.5, 22.5)	0 (0, 10)	15 (−11.08, 41.08)	0.074
Total spironolactone (mg)	0 (0, 125)	0	0 (−686.65, 686.65)	0.538
Total acetazolamide dose (mg)	0 (0, 375)	0	0 (−695.34, 695.34)	0.589
PaO_2_/FiO_2_ ratio at day 3	164 (143, 187)	135 (87, 190)	13 (−116, 143)	0.505
PaO_2_/FiO_2_ ratio at day 7	158 (105, 264)	151 (117, 184)	−14 (−178, 150)	0.945
PaO_2_/FiO_2_ ratio at day 10	121 (111, 227)	145 (112, 229)	−27 (−229, 174)	0.699
Change in SOFA score at day 3	−1.5 (−3.5, 1.5)	−1 (−2, 3)	0 (−6, 6)	0.670
Cumulative fluid balance at day 3 (mL)	−165.5 (−769, 280)	446 (−62, 760)	−944 (−2958, 1070)	0.161
Cumulative fluid balance at day 7 (mL)	−679 (−1491, 539)	−234 (−1859, 106)	−445 (−3020, 2130)	0.902
Cumulative fluid balance at day 10 (mL)	362 (−610, 853)	−360 (−388, 272)	243 (−2111, 2567)	0.699
Days alive and free of vasopressor at day 28	28 (27.5, 28)	21.5 (6, 26.5)	−6 (−28, 0)	0.01
Days alive and RRT-free at day 28	28 (28, 28)	28 (14, 28)	0 (0, 0)	1.000
Days alive and mechanical ventilation-free at day 28	8 (0, 19.5)	6 (0, 15)	4 (−13, 22)	0.769
ICU LOS (days)	24 (14.5, 56)	20.5 (12, 40)	8 (−45, 61)	0.721
Hospital LOS (days)	44 (30, 66.5)	38.5 (25, 61.5)	5 (−72, 82)	0.777
ICU mortality	1 (12.5)	3 (37.5)	0.33 (0.04, 2.56)	0.569
Hospital mortality	1 (12.5)	3 (37.5)	0.33 (0.04, 2.56)	0.569
28-d mortality	0	2 (25.0)	–	0.467
60-day mortality	1 (12.5)	3 (37.5)	0.33 (0.04, 2.56)	0.569

*AKI* acute kidney injury, *CI* confidence interval, *LOS* length of stay, *RRT* renal replacement therapy, *SOFA* Sequential Organ Failure Assessment, *ICU* intensive care unit

### Adverse events

There were numerically higher proportions of hypernatremia and metabolic alkalosis in the urine alkalinization group than in the control group (Table 4). There were no serious adverse events.

**Table 4:** Safety outcomes

	Intervention (n = 8)	Control (n = 8)	P value
Hypernatraemia, n (%)	6 (75)	4 (50)	0.608
Metabolic alkalosis, n (%)	8 (100)	5 (62.5)	0.200
Hypocalcaemia, n (%)	1 (12.5)	1 (12.5)	1.000
Hypokalaemia, n (%)	1 (12.5)	0	1.000

### Biomarker results and outcomes

There were 4 patients who developed AKI within 72 h after ICU admission, 3 of whom had AKI stage 2 or 3. Compared with the no AKI group or no AKI/AKI stage 1 group, patients who developed AKI had higher urine [TIMP-2]x[IGFBP7] results at day 0, 1 and 2 after ICU admission [Table S1 and Table S2].

None of the participants with urine [TIMP-2]x[IGFBP7] < 0.3 (ng/ml)^2^/1000 developed AKI. Twelve out of 16 patients had urine [TIMP-2]x[IGFBP7] ≥ 0.3 (ng/ml)^2^/1000 of whom 4 (33.3%) developed AKI (Table S3). All patients who developed AKI stage 2 or 3 had urine [TIMP-2]x[IGFBP7] concentrations ≥ 0.3 (ng/ml)^2^/1000 (Table S4). Patients with urine [TIMP-2]x[IGFBP7] ≥ 0.3 (ng/ml)^2^/1000 and AKI had a higher risk of receiving RRT and had the highest ICU and 60-day mortality (50%) (Table S5).

## Discussion

In this proof-of-concept feasibility RCT, urine alkalinization in critically ill patients with COVID-19 resulted in higher urine pH and more patients achieving urine pH ≥ 7.5 compared with the control group. Urine alkalinization was not associated with differences in changes in cell cycle arrest biomarkers. Other clinical outcomes including the incidence of AKI were not different between both groups. More patients in the intervention group experienced electrolyte and acid–base disturbances.

Coronaviruses including SARS-CoV-2 have been shown to require acidic conditions to enter cells [23]. Therefore, alkalinizing the local environment is considered a potential treatment for COVID-19. For example, nasal irrigation and oral rinses using sodium bicarbonate have been shown to reduce mortality, increase recovery rate, and reduce duration of hospitalization in COVID-19 patients albeit the studies were low quality [24].

Our protocol demonstrated an increase in urine pH in the alkalinization cohort, which occurred at 20 h and was sustained throughout the study period. The amount of sodium bicarbonate 8.4% (225 ml) and duration to achieve target urine pH were similar to previous data in the literature [25–27]. More patients in the intervention group experienced mild hypernatremia and mild metabolic alkalosis and we hypothesize that critically ill patients with COVID-19 with acute respiratory failure and high risk of AKI may be less metabolically compensated to sodium bicarbonate. The intervention group was more likely to receive diuretics, which may have influenced electrolyte concentrations and fluid balance. However, these metabolic derangements did not result in differences in fluid balance or oxygenation nor were any of these adverse effects considered severe.

Animal studies have shown the effects of urine alkalinization on the alleviation of drug-induced kidney injury [28–30]. Our study in patients with COVID-19 failed to demonstrate that the differences in urine pH were associated with differences in urine TIMP-2 and IGFBP7 concentrations. Twelve (75%) patients had urine [TIMP-2]x[IGFBP7] ≥ 0.3 (ng/ml)^2^/1000 indicating kidney stress. Further, patients with AKI had higher urine [TIMP-2]x[IGFBP7] results than those without AKI, and none of the patients with urine [TIMP-2]x[IGFBP7] < 0.3 (ng/ml)^2^/1000 developed AKI. Patients who had both, urine [TIMP-2]x[IGFBP7] ≥ 0.3 (ng/ml)^2^/1000 and AKI by creatinine criteria, experienced higher mortality than AKI patients with urine [TIMP-2]x[IGFBP7] < 0.3 (ng/ml)^2^/1000, suggesting that cell cycle arrest markers may have potential to prognosticate outcomes when combined with the conventional AKI criteria. This adds to the evolving data on the role of cell cycle arrest biomarkers in COVID-19. For instance, urine [TIMP-2]x[IGFBP7] results have been shown to predict moderate to severe AKI in patients with severe COVID-19 and acute respiratory distress syndrome [31].

To the best of our knowledge, this is the first RCT assessing the physiological effects of sodium bicarbonate and urine alkalinization in critically ill patients with COVID-19. There was a separation in urine pH and laboratory parameters between both groups which was sustained over time. We also investigated the predictive ability and prognostic potential of cell cycle arrest biomarkers between the groups.

We acknowledge significant limitations. First, as a proof-of-concept study, it was not powered to detect clinical differences. Second, we did not meet our planned recruitment target due to the low number of COVID-19 patients requiring ICU admission at the end of the pandemic. Third, we used an alkalinization protocol that had been established for other clinical conditions. We cannot be sure that alternative alkalinization methods would have resulted in different outcomes. Fourth, this was an open-label trial and there might have been co-intervention biases, such as fluid therapy, diuretics, hemodynamic management, use of nephrotoxic drugs, or electrolyte supplementation that impacted the outcomes.

## Conclusions

Urine alkalinization in critically ill patients with COVID-19 resulted in a sustained increase in urine pH, but there were no differences in clinical outcomes including AKI development. AKI patients with elevated urine biomarker concentrations had a higher risk of dying than AKI patients without a biomarker rise. Larger studies are needed to explore the relationship between urine alkalinization and the development and severity of AKI in patients with COVID-19.

## Supplementary Information

Additional file 1

## Data Availability

The datasets used and/or analyzed during the current study are available from the corresponding author on reasonable request.

## Abbreviations

ACE2: Angiotensin-converting enzyme-2
ACEI: Angiotensin-converting enzyme inhibitor
AKI: Acute kidney injury
APACHE: Acute physiology and chronic health evaluation
ARB: Angiotensin receptor blocker
BMI: Body mass index
CI: Confidence interval
CKD: Chronic kidney disease
COPD: Chronic obstructive pulmonary disease
COV-AKI: Acute kidney injury secondary to Coronavirus disease 2019
COVID-19: Coronavirus disease 2019
CRP: C-reactive protein
CV: Coefficient of variation
Hb: Hemoglobin
HRA: Health research authority
ICU: Intensive care unit
IGFBP7: Insulin-like growth factor-binding protein 7
IQR: Interquartile range
ITT: Intention-to-treat
KDIGO: Kidney disease improving global outcomes
LOS: Length of stay
MHRA: Medicines and healthcare products regulatory agency
NSAID: Non-steroidal anti-inflammatory drugs
RBD: Receptor binding domain receptor binding domain (RBD)
RCT: Randomized controlled trial
REC: Research ethics committee
RR: Risk ratio
RRT: Renal replacement therapy
RTEC: Renal tubule epithelial cells
SARS-CoV-2: Severe acute respiratory syndrome coronavirus 2
SCr: Serum creatinine
SOFA: Sequential organ failure assessment
TIMP-2: Tissue inhibitor of metalloproteinases-2
TMPRSS2: Transmembrane protease serine 2
WBC: White blood cells
