# Supplementary material for: The effects of urine alkalinization on kidney function in critically ill patients with COVID-19: a proof-of-concept randomized clinical trial
Source: Intensive Care Med Exp. 2025 Mar 7;13:33. doi: 10.1186/s40635-025-00739-7 (PMC11889288; doi:10.1186/s40635-025-00739-7)
Supplement: Supplementary file 1 — Additional file 1 [file 40635_2025_739_MOESM1_ESM.docx]

**Supplemental material**

**Table S1 Concentrations of urine [TIMP-2]x[IGFBP7]* in patients with AKI within 72 hours**

| Day of measurement | AKI (n=4) | No AKI (n=12) | P value |
| --- | --- | --- | --- |
| 0 | 1.46 (0.37, 2.47) | 0.29 (0.16, 0.59) | 0.210 |
| 1 | 0.56 (0.28, 4.77) | 0.19 (0.09, 0.86) | 0.180 |
| 2 | 0.51 (0.39, 0.47) | 0.21 (0.14, 0.50) | 0.088 |

**Abbreviation:** AKI, acute kidney injury

* in (ng/ml)^2^/1000

**Table S2 Concentrations of urine [TIMP2]x[IGFBP7]* in patients with AKI stage 2-3 within 72 hours**

| Day | AKI stage 2/3 (n=3) | No AKI/AKI stage 1 (n=13) | P value |
| --- | --- | --- | --- |
| 0 | 2.29 (0.12, 2.64) | 0.3 (0.18, 0.62) | 0.296 |
| 1 | 0.42 (0.28, 0.56) | 0.2 (0.09, 1.02) | 0.686 |
| 2 | 0.93 (0.39, 1.47) | 0.27 (0.16, 0.51) | 0.198 |

**Abbreviation:** AKI, acute kidney injury

* in (ng/ml)^2^/1000

**Table S3 Urine [TIMP-2]x[IGFBP7] concentrations in patients with and without AKI in first 72 hours**

| AKI status | urine [TIMP-2]x[IGFBP7] < 0.3 (ng/ml)^2^/1000 (n=4) | urine [TIMP-2]x[IGFBP7] ≥ 0.3 (ng/ml)^2^/1000 (n=12) | P value |
| --- | --- | --- | --- |
| No AKI | 4 (100) | 8 (66.7) | 0.516 |
| AKI | 0 | 4 (33.3) |  |

**Abbreviation:** AKI, acute kidney injury

**Table S4 Urine [TIMP-2]x[IGFBP7] concentrations in patients with and without moderate to severe AKI in first 72 hours**

| AKI status | urine [TIMP-2]x[IGFBP7] < 0.3 (ng/ml)^2^/1000 (n=4) | urine [TIMP-2]x[IGFBP7] ≥ 0.3 (ng/ml)^2^/1000 (n=12) | P value |
| --- | --- | --- | --- |
| No AKI or AKI stage 1 (n=13) | 4 (100) | 9 (75) | 0.529 |
| AKI stage 2/3  (n=3) | 0 | 3 (23) |  |

**Abbreviation:** AKI, acute kidney injury

**Table S5 Comparison of outcomes in patients with positive or negative Nephrocheck result and AKI status at 72 hours**

| Group | NC-/AKI-  (n=4) | NC+/AKI-  (n=8) | NC-/AKI+ (n=0) | NC+/AKI+  (n=4) | P value |
| --- | --- | --- | --- | --- | --- |
| RRT | 0 | 0 | - | 2 (50) | 0.100 |
| ICU LOS | 45 (32, 57) | 18 (14, 37) | - | 19 (6, 32) | 0.203 |
| Hospital LOS | 56 (41, 90) | 48 (23, 68) | - | 33 (18, 37) | 0.178 |
| ICU mortality | 1 (25) | 1 (12.5) | - | 2 (50) | 0.508 |
| 60-day mortality | 1 (25) | 1 (12.5) | - | 2 (50) | 0.508 |

**Abbreviation:** AKI, acute kidney injury; NC-, Nephrocheck result [TIMP-2]x[IGFBP7] <0.3 (ng/ml)^2^/1000; NC+, Nephrocheck result [TIMP-2]x[IGFBP7] >0.3 (ng/ml)^2^/1000; RRT, renal replacement therapy; ICU, intensive care unit; LOS, length of stay
